# Functional Study of *NIPA2* Mutations Identified from the Patients with Childhood Absence Epilepsy

**DOI:** 10.1371/journal.pone.0109749

**Published:** 2014-10-27

**Authors:** Han Xie, Yuehua Zhang, Pingping Zhang, Jingmin Wang, Ye Wu, Xiru Wu, Theoden Netoff, Yuwu Jiang

**Affiliations:** 1 Department of Pediatrics, Peking University First Hospital, Beijing, P.R. China; 2 Department of Biomedical Engineering, University of Minnesota, Minneapolis, Minnesota, United States of America; Cambridge University, United Kingdom

## Abstract

Recently many genetic mutations that are associated with epilepsy have been identified. The protein NIPA2 (non-imprinted in Prader-Willi/Angelman syndrome region protein 2) is a highly selective magnesium transporter encoded by the gene *NIPA2* in which we have found three mutations (p.I178F, p.N244S and p.N334_E335insD) within a population of patients with childhood absence epilepsy (CAE). In this study, immunofluorescence labeling, inductively coupled plasma-optical emission spectroscopy (ICP-OES), MTT metabolic rate detection and computational modeling were utilized to elucidate how these mutations result in CAE. We found in cultured neurons that NIPA2 (wild-type) proteins were localized to the cell periphery, whereas mutant proteins were not effectively trafficked to the cell membrane. Furthermore, we found a decrease in intracellular magnesium concentration in the neurons transfected with mutant *NIPA2*, but no effect on the survival of neurons. To understand how low intracellular magnesium resulted in hyperexcitability, we built and analyzed a computational model to simulate the effects of mutations. The model suggested that lower intracellular magnesium concentration enhanced synaptic N-methyl-D-aspartate receptor (NMDAR) currents. This study primarily reveals that a selective magnesium transporter NIPA2 may play a role in the pathogenesis of CAE.

## Introduction

Epilepsy is a collection of neurologic diseases characterized by unprovoked and recurrent seizures. Childhood absence epilepsy (CAE) is considered a crucial type of genetic generalized epilepsy (GGE). Previous studies have only found mutations in ion channel genes associated with CAE, such as *CACAN1H* [1]–[3], which encodes a T-type Ca^2+^ channel. Recently, some non-ion channel genes leading to the CAE have also been identified, such as *NIPA2* [4]. *NIPA2* is located at 15q11.2, a region associated with GGE. In our previous study, we found three *NIPA2* mutations in a population of patients with CAE. They included two novel missense mutations (c.532A>T, p.I178F; c.731A>G, p.N244S) and one novel small insertion (c.1002_1003insGAT, p.N334_E335insD) [4].

*NIPA2* encodes the non-imprinted in Prader-Willi/Angelman syndrome region protein 2 (NIPA2) [5]. NIPA2 consists of 360 amino acids and has 9 transmembrane protein domains. It belongs to the NIPA family of proteins. The NIPA family members are integral membrane proteins which function as magnesium transporters and include NIPA1, NIPA2, NIPA3 and NIPA4 [6], [7]. NIPA1, NIPA3 and NIPA4 transport Mg^2+^ as well as other cations. NIPA2 is a highly selective magnesium transporter located in the cytomembrane and the early endosome [7]. Its function is to transfer extracellular Mg^2+^ into the cytoplasm [8]. To date, no functional study about *NIPA2* mutations has been reported. Functional studies from *NIPA1* mutations have shown that mutations disrupt transport of the protein to the extracellular membrane, resulting in an accumulation of the proteins in the cytoplasm [9]. Based on this finding we hypothesize that *NIPA2* mutations also affect transport, resulting in accumulation of the proteins in the cytoplasm and a decreased intracellular Mg^2+^ concentration.

Mg^2+^ participates in the gating and activation of channels and receptors, such as N-methyl-D-aspartate receptors (NMDARs) [10], [11], which play a role in synaptic plasticity [12], [13]. Absence epilepsy is thought to be generated by pathological behavior in the thalamocortical loop [14]. NMDARs are widely distributed among excitatory neurons in the thalamus and cortex. Therefore, it is easy to imagine how decreased intracellular Mg^2+^ concentration caused by the *NIPA2* mutation may result in epilepsy.

## Results

### The accumulation of NIPA2 mutant proteins in cytoplasm

In a previous genetic study, we discovered three mutations in the *NIPA2* Mg^2+^ transporter gene associated with a form of childhood absence epilepsy [4]. Studies of *NIPA1* mutations found that the mutations prevented transport of the NIPA1 protein to the extracellular membrane, resulting in an accumulation of the proteins in the endoplasmic reticulum (ER) [6]. NIPA2 was also responsible for transport of Mg^2+^ [8]. Production of NIPA2 was Mg^2+^ dependent; when extracellular Mg^2+^ was low the cell compensated with a significant increase in membrane expression of NIPA2 [7], presumably to restore the intracellular concentration to normal. Based on these findings, we hypothesized that decreased functional NIPA2 in the extracellular membrane would result in decreased intracellular Mg^2+^ concentration. In this study, we transfected primary cultured neurons with three different *NIPA2* mutations (two novel missense mutations: p.I178F and p.N244S; one novel small insertion: p.N334_E335insD) and wild-type with HSV-RFP vectors (Herpes Simplex Virus - Red Fluorescence Protein).

**Figure 1:**
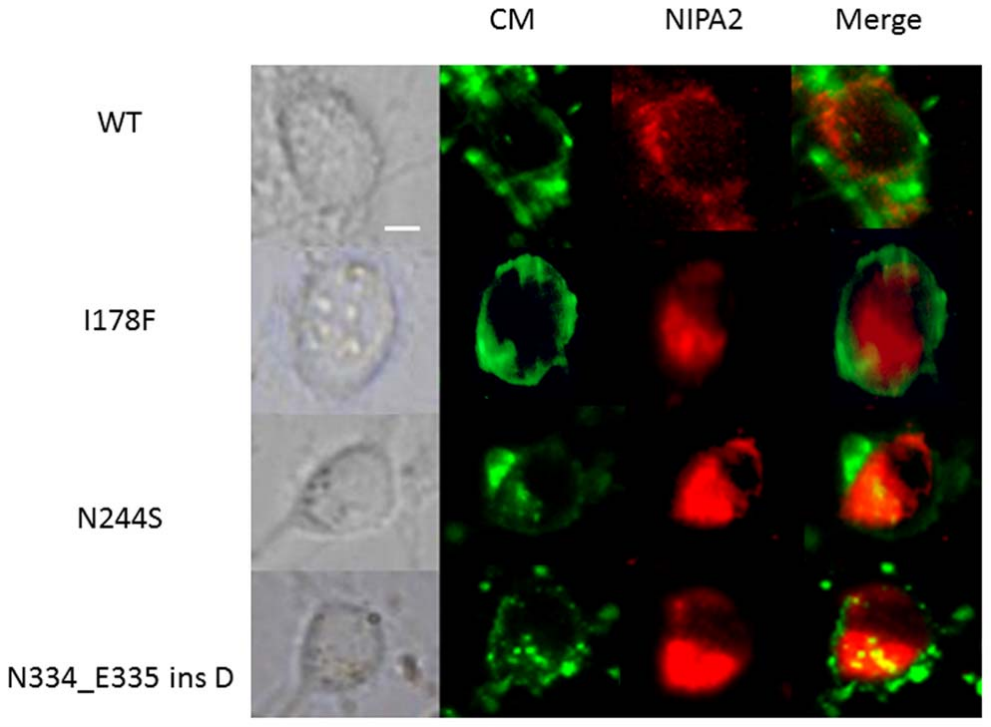
The localization of NIPA2-WT and the mutants in cultured neurons. CM: cytomembrane marked by DIO (a cytomembrane marker, green); NIPA2 was labeled by red fluorescence. MERGE: overlapped image. WT: wild-type. NIPA2-WT proteins (red) were distributed at cytomembrane, overlapped with DIO (green), but the three mutant proteins (I178F, N244S, N334_E335insD; red) were trapped in cytoplasm and showed no overlap with the plasma membrane. n = 4 experiments, scale bar: 5 µm.

The first goal was to determine the location of the mutant NIPA2 proteins expressed in the cells. To do this, they were marked with red fluorescence protein (RFP) and the cell's plasma membrane was labeled with a green fluorescence marker (DIO). We found that wild-type proteins were localized to the cell border, but all three mutant proteins were retained in the cytoplasm (Figure 1).

### Cell viability of transfected neurons

A number of proteins accumulating in cytoplasm, especially in the endoplasmic reticulum (ER), may cause apoptosis through the unfolded protein response (UPR) and ER stress [15]. Therefore, we hypothesized that NIPA2 proteins trapped in the cytoplasm would influence cell viability. In this study, MTT metabolic rate detection was performed to observe whether cell viability of cultured neurons with *NIPA2* mutations decreased. MTT (3-(4, 5-dimethylthiazol-2-yl)-2, 5-diphenyltetrazolium bromide) is a yellow substrate that is enzymatically cleaved in living cells to yield a dark blue formazan product. This process requires active mitochondria, and cells that have recently died do not cleave significant amounts of MTT. Primary cultured neurons were transfected with the wild-type *NIPA2*, three *NIPA2* mutants and the *NIPA2*-siRNA. Cell viability of transfected cells was detected by fluorescence measured with an ELISA plate reader 36–48h after the transfection.

**Figure 2:**
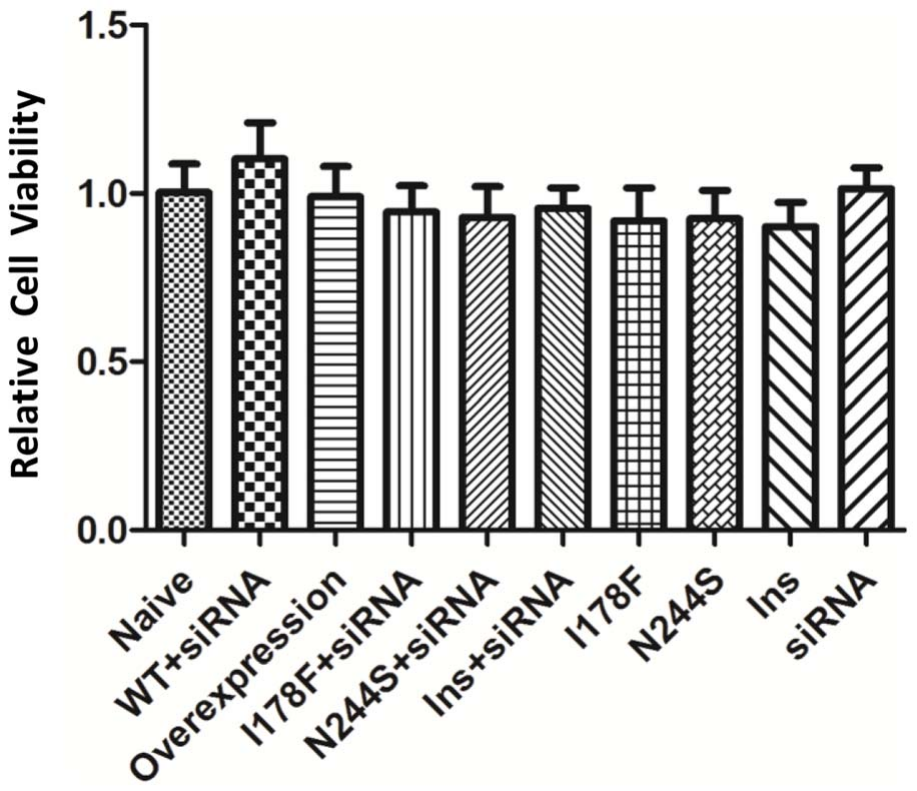
Cell viability of neurons with wild-type or mutant *NIPA2*. Cell viability was measured by MTT detection. The absorbance of formazan was proportional to metabolic activity of the cells. Relative cell viability was calculated as: (mean absorbance of transfected group - mean absorbance of basal temperature control)/(mean absorbance of naïve group - mean absorbance of basal temperature control). Naïve: the cultured neurons without transfection; Overexpression: neurons transfected with *NIPA2* ^WT^; Ins: the small insertion (N334_E335insD); siRNA: neurons transfected with *NIPA2*-siRNA. Neurons were co-transfected with the mutant and *NIPA2*-siRNA in the groups (I178F+siRNA, N244S+siRNA and ins+siRNA). The group (WT+siRNA) represented the neurons transfected with *NIPA2* ^WT^ and *NIPA2*-siRNA. The mutant groups (I178F, N244S and Ins) represented the neurons transfected only with the mutant. n = 9 experiments. No significant differences between naïve group and any other condition were found.

The naïve cultured neurons were compared to: the overexpression group, in which exogenous wild-type NIPA2 was added to the endogenous NIPA2 proteins; the WT+siRNA neurons, which were co-transfected with an exogenous copy of the wild-type *NIPA2* and the siRNA to knock down the NIPA2 expression to achieve approximately the normal concentration of the NIPA2; the mutant+siRNA groups, such as the mutant I178F are transfected with the siRNA to promote the mutant and suppress the endogenous protein; and the mutant-only group expressing I178F, N244S, or the small insertion protein, which is expressed along with the endogenous protein; and finally, siRNA alone, to determine the effect of low endogenous NIPA2 expression. There was no significant difference among the groups (analyzed by Prism 5.0, One-way ANOVA, p = 0.8433) (Figure 2).

### Intracellular Mg^2+^ concentrations decreased in neurons with mutations

**Figure 3:**
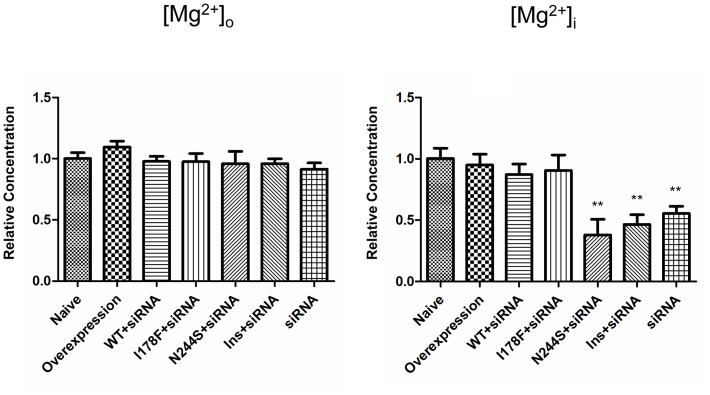
The concentration of extra/intracellular Mg^2+^ measured by ICP-OES. There was no significant change of extracellular Mg^2+^ concentration among the groups compared to naïve cells; the mutant N244S and Ins dramatically decreased the concentration of intracellular Mg^2+^, though the mutant I178F and the overexpression group were not different from the naïve group. Naïve: the cultured neurons without transfection; Overexpression: neurons transfected with *NIPA2* (WT); Ins: N334_E335insD. siRNA: neurons transfected with *NIPA2*-siRNA; the group (WT+siRNA): neurons transfected with *NIPA2* ^WT^ and *NIPA2*-siRNA; the mutant+siRNA group (I178F+siRANA, N244S+siRNA, Ins+siRNA): neurons transfected with the mutant and *NIPA2*-siRNA. Relative concentration was calculated as: Mg^2+^ concentration of the transfected group/Mg^2+^ concentration of naïve group. n = 4 experiments.

The accumulation of NIPA2 proteins in cytoplasm did not affect cell viability, but due to the location of the proteins in the cytoplasm, we hypothesized that it might decrease intracellular Mg^2+^ concentration. The four members of the NIPA family all are Mg^2+^ transporters; however, only NIPA2 is highly selective for Mg^2+^ [7]. NIPA2 plays a vital role in Mg^2+^ influx [8], [16], and incorrect localization may decrease the intracellular Mg^2+^ concentrations significantly. To test this, we applied inductively coupled plasma-optical emission spectroscopy (ICP-OES) to measure extracellular/intracellular Mg^2+^ concentration. Results were shown in Figure 3. For the missense mutant I178F cells we found a small but insignificant decrease on intracellular Mg^2+^ concentration. However, for the missense mutant N244S and the small insertion (N334_E335insD) mutation there was a significant decrease in the concentration of intracellular Mg^2+^ by 62% and 53% respectively (analyzed by Prism 5.0, One-way ANOVA, *p = 0.0003*). Compared to the naïve group the N244S+siRNA (t-test, *p* = 0.0066. **), Ins+siRNA (*p* = 0.0034, **), and siRNA (*p* = 0.0046, **) had significantly lower intracellular Mg^2+^ concentration, but the group I178F+siRNA did not (*p* = 0.5435). In no case did the mutations significantly alter the extracellular Mg^2+^ concentration (Prism 5.0, One-way ANOVA, *p* = 0.5414).

### Modeling: Effects of low intracellular Mg^2+^ on NMDA receptor currents

**Figure 4:**
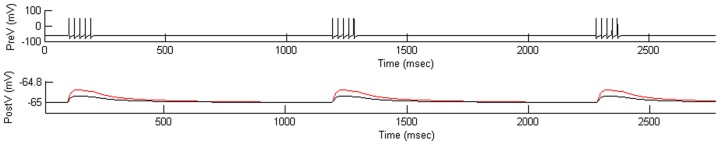
The effect of low intracellular Mg^2+^ on NMDAR model. PreV: the voltage of the presynaptic neuron, induced to fire in three bursts of action potentials; PostV: the postsynaptic potential from NMDA synaptic current in response to input from presynaptic cell above. Black trace was the response in normal intracellular Mg^2+^ (1 mM) and the red trace was the response in low intracellular Mg^2+^ (0.1 mM). The amplitude of postsynaptic potential increased as the intracellular Mg^2+^ concentration dropped to one tenth the normal value by unblocking the NMDAR current.

We hypothesized that low intracellular Mg^2+^ may affect the NMDAR currents. To test this hypothesis, we used an NMDAR synaptic model [17], [18] and modified it to simulate the effects of intracellular Mg^2+^. The NMDAR was blocked by intracellular Mg^2+^ [19]; decreasing intracellular Mg^2+^ increased the NMDAR currents. A simulation of the post-synaptic conductance during three bursts of synaptic inputs in normal and low intracellular Mg^2+^ was shown in Figure 4. When intracellular Mg^2+^ was set at one-tenth the normal value (0.1 mM), the amplitude of postsynaptic potential during the tonic phase of the burst was increased (Figure 4).

## Discussion

NIPA2, a highly selective Mg^2+^ transporter, may play an important role in the blocking of NMDARs via the regulation of Mg^2+^ concentration, and thus has a vital effect on the excitability of neural network excitability. Our previous genetic study was the first to identify three mutations in Chinese patients with CAE [4]. From these findings, we infer that *NIPA2* mutations may affect neural network behavior. However, no functional studies have been done to understand how *NIPA2* mutations result in CAE. To reveal the mechanism by which *NIPA2* mutations cause CAE, we measured cell viability, protein localization, and extracellular/intracellular Mg^2+^ concentration in neuronal cultures. We then measured cell excitability in computational models. In a protein localization assay, we found that *NIPA2* mutations led to the incorrect localization of the NIPA2 protein in neurons, resulting in a decrease of intracellular Mg^2+^. In computational models, we inferred that a decrease in intracellular Mg^2+^ concentration enhanced NMDAR currents, putatively resulting in the pathogenesis of CAE.

### Incorrect protein localization affects Mg^2+^ transport in transfected neurons with *NIPA2* mutations

Due to the function of NIPA2, i.e. the transportation of Mg^2+^ into the cytoplasm [8], [16], we have predicted that incorrect protein localization may decrease the concentration of intracellular Mg^2+^. In this study, we have observed the significant decrease in intracellular Mg^2+^ in a mutant model (N244S, N334_E335insD) and a knock-down model, while the concentration of extracellular Mg^2+^ was not changed significantly (within the detection precision of ICP-OES).

However, decreased intracellular Mg^2+^ was not seen in all mutants. No significant change in intracellular Mg^2+^ concentration was detected in the neurons with the I178F mutation, yet these mutant proteins were retained in cytoplasm as the neurons with other mutants. In a previous study it was found that *NIPA1* mutant proteins were distributed in the cytomembrane but its function in Mg^2+^ transport was diminished [7]. We infer that in some of the mutations both the distribution of the protein and Mg^2+^ transport are affected, while in the mutant I178F only the transport is affected. In the mutant I178F, the small amount of protein in the cytomembrane is able to effectively transport Mg^2+^ into the neuron. Some protein accumulation in the neurons with the mutant I178F may not significantly affect intracellular Mg^2+^ concentration. Therefore, we believe that while the I178F mutation may inhibit NIPA2 protein trafficking to cell membranes, it does not also affect the function at the cell membrane. In contrast N244S and N334_E335insD decrease both the cytomembrane concentration and Mg^2+^ transport.

### *NIPA2* mutations did not affect cell viability of transfected neurons

NIPA1, another member of NIPA family, is similar to NIPA2 in terms of biological features. NIPA1 is a transmembrane protein, and also functioned as an Mg^2+^ transporter [20]. Earlier studies of NIPA1 mutant proteins found that they accumulated in the ER and caused cellular toxicity [21]. Thus we tested whether neurons with *NIPA2* mutations encountered the same problems. In this study, mutant NIPA2 proteins trapped in cytoplasm were observed; however, the accumulation of mutant proteins did not affect the viability of the neurons. We infer that the degree of accumulation caused by *NIPA1* mutations may be more serious than that caused by *NIPA2* mutations. The apoptosis resulting from excessive UPR and ER stress in the cells with *NIPA1* mutations may not occur in the transfected neurons with *NIPA2* mutations. Thus we have found no significant change in viability in the transfected neurons with *NIPA2* mutations.

### *NIPA2* mutations may enhance NMDAR currents

Mg^2+^ participates in the gating and activation of channels and receptors, such as NMDARs [10], [11], which play a role in modulations of neural excitability [12], [13]. Intracellular Mg^2+^ blocks the activation of NMDAR channels to modulate synaptic strength. Based on our findings that *NIPA2* mutations decrease intracellular Mg^2+^, we hypothesize that the mutations may cause enhanced NMDAR currents. In our computational model, we have observed that low intracellular Mg^2+^ increases NMDAR-related synaptic currents significantly. It supports our hypothesis intracellular Mg^2+^ increases NMDAR currents, But further electrophysiological experiments are needed to test this theory. Since there is not a specific blocker of the Mg^2+^ transporter to build a model of low intracellular Mg^2+^ for extracellular recording, computational modeling has been used in this study to test the effect of low intracellular Mg^2+^. We believe a mutant animal model may be ideal and needed to further study the effect of these mutations.

### *NIPA2* mutations may contribute to childhood absence epilepsy

We have found the three mutations lead to functional changes, suggesting they may be pathogenic. The *NIPA2* mutations may play a role in the pathogenesis of CAE. CAE is known as a multigenic disease. *CACAN1H* and other genes associated with CAE have been reported [1]–[3]. Each of them may increase risk for the development of CAE. *NIPA2* is no exception. The three *NIPA2* mutations identified in our previous study support this issue [4]. However, Hildebrandt and his colleagues have not found any *NIPA2* mutations in a large Caucasian cohort [22]. We suppose different frequencies between the Caucasian and Chinese population and low incidence may account for the negative result of Hildebrandt's study [23]. In addition, only typical CAE patients were involved in our previous study, but several forms of GGE were included in Hildebrandt's study. The inconsistency of the cases is probably another reason why they have obtained a negative result [23]. We believe further studies with Caucasian and other populations are needed to profoundly discuss the association between *NIPA2* mutation and CAE.

### Summary

We demonstrate that NIPA2 mutant proteins accumulate in the cytoplasm, lowering intracellular Mg^2+^. Low intracellular Mg^2+^ concentration may enhance NMDAR currents. This study describes functional changes of a highly selective Mg^2+^ transporter *NIPA2* mutations. This study may provide insights to the elaboration of pathogenic mechanism and the development of further treatments for childhood absence epilepsy.

## Materials and Methods

### Ethics statement

Primary cultured neurons were prepared from pregnant Sprague-Dawley (SD) rats at the gestational age of 16–18 days. The rats were used in accordance with protocols approved by the experimental animal sciences of Peking University Health Science Center Institutional Animal Care and Use Committee (IACUC).

### Cell culture

Primary cultured neurons were prepared from pregnant SD rats at the gestational age of 16–18 days. The procedures were all in accordance with ARRIVE guidelines (http://www.nc3rs.org.uk/page.asp?id=1357). Pregnant SD rats were anaesthetized by intraperitoneal injection of 10% chloral hydrate, and fetal rats were obtained by the cesarean. The fetal cerebral cortices were dissected with 0.5 mM EDTA and 0.5 mM cysteine-HCl (Sigma) in Earle's balanced salt solution, and then the cortices were gently triturated. The triturated cortices were digested at 37°C for 15–25 min with 1 mg/ml papain (0.5–2 units/mg, Sigma) before dissociated cells were suspended in the plating medium (Minimum Essential Medium containing 10% fetal bovine serum, 100 IU/ml penicillin and 100 IU/ml streptomycin). The number of dissociated cells was counted. 2–5 ml of cell suspension was planted on the coverslips (Sigma) in 24-well plates at a density of 1.5–3.0×10^5^ cells/ml. Cells were incubated at 37°C in a 95% O_2_, 5% CO_2_ humidified incubator for 4–6 h. Then the plating medium was replaced with the culture medium (Neurobasal A (Gibco BRL) medium containing 2% B27 and 0.5 mM L-glutamine (Gibco BRL)). The culture medium was replaced every 3–4 days.

### Constructs and transfections of *NIPA2* HSV vectors

Different groups of cultured neurons were transfected with *NIPA2* (wild-type), *NIPA2*-siRNA, missense mutants (I178F, N244S) and a small insertion (N334_335EinsD) (MOI value = 1.5). Herpes simplex virus (HSV) vectors with *NIPA2* (wild-type, mutations or siRNA) were conducted by OrienGene Biotechnology Ltd. (Beijing, China). To identify transfected proteins all vectors were labeled with red fluorescent protein (RFP). The concentrated liquid containing the vectors was diluted by culture medium Neurobasal A (Gibco BRL) medium containing 2% B27 and 0.5 mM L-glutamine (Gibco BRL). The diluted liquid was added to coverslips with cultured neurons, and then the coverslips were incubated at 37°C in a 95% O_2_, 5% CO_2_-humidified incubator. The medium was replaced after the first 24 h, and every 3 days thereafter. Transfected cells were either processed for MTT testing, immunofluorescence labeling, or Mg^2+^ concentration measurement.

### Immunofluorescence

To observe the localization of NIPA2 proteins, cultured cells were washed with 0.1M phosphate-buffered saline (PBS) three times before being fixed in 4% paraformaldehyde for 10 min. After that, the cells were incubated with DIO (1∶500; Abcam), a specific cytomembrane marker, for 40 min at room temperature. After washing with PBS, stained coverslips were observed by a fluorescent microscope (DMIRB, Leica, Germany). The steps were all performed at room temperature.

### Cell viability test

Viability of the transfected cells was measured by the MTT (3-(4, 5-dimethylthiazol-2-yl)-2, 5-diphenyltetrazolium bromide) assay as described by Hussain et al. [24] with minor modifications. The MTT assay (Cellchip Biotechnology, Beijing, China) was performed on the naïve group and on transfected groups. MTT (50 µl per hole) was added in 24-well plates with cultured cells. After cells were incubated for 4 h, formazan solution was added to the plates. The absorbance of formazan was measured at 570 nm by an enzyme-linked immunosorbent assay (ELISA) plate reader (Bio-Rad Model 550, USA), and the relative cell viability was calculated as: 
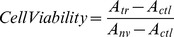



where *A_tr_* is the mean absorbance of cells in the transfected group, *A_ctl_* is the mean absorbance of the basal temperature control, and *A_nv_* is the mean absorbance of the naïve group.

### Inductively coupled plasma mass spectrometry optical emission spectrometry

Extracellular/intracellular Mg^2+^ was measured by the inductively coupled plasma mass spectrometry optical emission spectrometry (ICP-OES). The culture medium was collected to measure extracellular Mg^2+^. 1 ml culture medium of each group was sent to the department of toxicology (Peking University Health Science Center, Beijing, China) for ICP-OES measurement. The extracellular Mg^2+^ concentration was calculated by mg/ml. Plated cells were washed by the phosphate buffer saline (PBS) medium (which was Mg^2+^ free) to purge residual culture medium at the surface of plated cells, and were then scraped in a small amount of PBS medium and collected into centrifuge tubes. The medium with cells was centrifuged at 3000 rpm for 10 min. The cell mass pellet was air dried and the cell mass was weighed. The cell mass was then sent to the department of toxicology for ICP-OES measurement. Mg^2+^ concentration of each group was measured by the plasma atomic emission spectroscopy (ICAP 6000, Thermo, UK). The testing conditions were as follows: incident power 1.15 KW, auxiliary gas 0.5 L/min, observation height 12 mm, wavelength 279.6 nm, detection limit 0.91 ng/ml. The Mg^2+^ concentration of the cell mass was used as an estimate of the concentration of intracellular Mg^2+^ [25]. The intracellular Mg^2+^ concentration of each group was normalized as: 
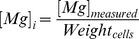



where [*Mg*]*_measured_* is the Mg^2+^ concentration measured by ICP-OES, and *Weight_cells_* is the weight of the cell mass of each group.

### Computational modeling: NMDAR model with intracellular Mg^2+^ dependence

The NMDAR glutamate receptor played a vital role in synaptic plasticity and development as well as epilepsy. NMDAR was activated by glutamate and either D-serine or glycine and was blocked by extracellular and intracellular Mg^2+^ [10], [11], [19]. To simulate the effects of intracellular Mg^2+^ block of NMDAR receptors [19], we modified an NMDAR model by Destexhe et al. [17], [18] from MODEL DB. In Destexhe's model, the NMDAR channel was blocked by extracellular Mg^2+^ but not intracellular Mg^2+^. To incorporate the influence of intracellular Mg^2+^ concentrations on NMDAR synaptic transmission, we modified a term in the model to account for the intracellular and extracellular Mg-block. The equation describing Mg-block in the NMDAR model was as follows:
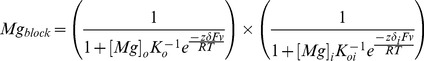



where *K_oi_* of intracellular Mg^2+^ = 3.4 [26], *δ_i_* of intracellular Mg^2+^ = 0.95 [19],  = −0.0668, and the definition of the components were defined in Destexhe's model [17], [18].

The effect of Mg^2+^ block on NMDAR conductance *G_NMDA_* was modeled as follows [17], [18]:
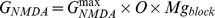



where 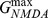 is the maximal conductance, and *O* is the proportion of ligand-gated channels that are open [17], [18].
